# Sustainability Transitions in E-commerce Research—Academic Achievements and Impediments

**DOI:** 10.1007/s43615-023-00252-7

**Published:** 2023-01-11

**Authors:** Mengzhen Zhang

**Affiliations:** Department of Forest Sciences, University of Helsinki, 00014 Helsinki, Finland

**Keywords:** E-commerce, Sustainability transitions, Governing transitions, Literature review

## Abstract

To date, the COVID-19 pandemic has led to the blossoming of e-commerce, which has brought both advantages and impediments to a more sustainable future. The central aim of sustainability transitions (ST) research conceptualizes and explains how radical changes can occur in the way that societal and environmental functions are fulfilled. Embedding ST logic with e-commerce could help us understand the current standing of e-commerce, and lead to solutions applied from its implications. However, there is a lack of research that pivots ST into the context of e-commerce. Thus, this paper fills the gap by conducting a comprehensive literature review to look into how the current e-commerce research fits into the ST framework. We find that the current sustainable e-commerce research is unevenly scattered alongside different dimensions, and there is an urgency to employ government power and drive public awareness. This paper extends the scope of ST into the e-commerce context; solutions for practitioners to achieve effective governance have been particularly emphasized.

## Introduction

E-commerce evolves and plays a significant role in the digital market, representing the main mechanism for implementing the new type of economy [1]. The proven positive effects and continued growth of e-commerce have not only attracted substantial attention among various research disciplines for several years, but also gained ample success in various industries. For example, e-commerce boosts the circulation of agricultural products and promotes market transparency and price discovery [2, 3], reduces supplier costs in the supply chain, and enhances transaction efficiency [4]. Besides, e-commerce is also considered to have a broader positive impact on nearly everyone with access to the Internet [5], providing such distinctive advantages as rich selection variety, more competitive prices, shopping 24 h a day, and geographical location independence. E-commerce is gradually becoming the primary first choice for consumers to go shopping. Additionally, the unprecedented COVID-19 pandemic has brought inexorable growth to e-commerce [6], which has led to e-commerce gaining an overwhelming and ever-expanding market share. As a result of the COVID-19 impact, on the one hand, consumers are increasingly purchasing online, while the business landscape has also been altered, leading to more online supply to reduce in-person contact [7]. On the other hand, during the pandemic and implementation of preventive measures, e-commerce also caused the volume of transport and distribution to increase by more than 100% in some cities and therefore brought a significant ecological impact and presented an unsustainable development [8].

Even though emerging e-commerce is considered to have great potential for positive impacts on the environment, each positivity is coupled with a potentially negative impact also [9]. Therefore, understanding in which direction e-commerce is now heading and what advantages and deficiencies e-commerce has is of great importance for us to understand the sustainable promises that e-commerce could bring us. Analytical academic research is therefore needed to summarize the current stage and foresee possible directions where sustainable e-commerce can lead us.

However, as far as we are aware, less research attention has been given to how e-commerce achieves sustainability in a highly competitive and low-margin retailing sector [10]. There exists limited theoretical research that focuses on existing academic works of literature to understand which stage sustainable e-commerce (SE) is in, not to mention to draw conclusions and propose possible directions regarding its future development. Besides, there remains a lack of a comprehensive framework to understand how the e-commerce environment [11] fits into the previously mentioned complicated settings. Therefore, to bridge the abovementioned gap, we conduct a literature review work to investigate what are the current research trends regarding e-commerce sustainability. Literature review is a useful approach to summarize past research outcomes and to further understand where the academic discussion might be going. Scopus database is selected for a scan of the published academic papers as it offers refining features and sorting for researchers to acquire research works that dated back to the mid-1960s [12]. It also provides more coverage than other databases, e.g., Web of Science [13]. To achieve such a goal, this paper is formulated to answer three research questions:

- (RQ1)What kinds of research themes in the domain of SE have been discussed?
- (RQ2)Which are the most frequently discussed topics and which are the least when integrating the ST framework with SE literature?
- (RQ3)What themes are there lacking in the current scientific papers and what future directions might be suggested from this study?

The structure of this paper is designed to answer the above-mentioned questions. In the following “Theoretical Background” section, ST concepts are introduced, followed by illustrating major themes and prevailing theories in the existing literature. A classificatory tool is also designed to answer the presented questions. The paper ends with solutions and managerial implications drawn from this analysis, followed by suggesting future research directions, with greater attention paid to policy implementation and government engagement.

## Theoretical Background

### Sustainability Transitions

The term “sustainability transitions” originated from “transition,” which appeared broadly in multiple scientific disciplines, for example, ecology and psychology. The initial focus of transitions research is mainly on understanding persistent challenges in the socio-technical system and probing to present solutions to sustain natural resources and biodiversity; one well-known example is energy transition. Keywords that have been frequently used in the early stage to reach energy efficiency and resource conservation are, for example, clean technology, chemical-specific regulations, and environmentally compatible materials. Environmentally beneficial technologies especially the possibilities of inducing large-scale technological transitions into achieving a sustainable economy is the mainstream of the research focus in the 1990s [14]. From then on, the research scope is also extended to a wider societal and political sphere due to the arising awareness that technology alone is insufficient to achieve such fundamental and radical changes; integrating policy approaches for a long-term transition is crucial to respond to such systemic challenges [15]. At the end of the 1990s, an inter- and transdisciplinary research field emerged so as to partake and adapt to transitions to come and accelerate sustainable development—which, therefore, refers to sustainability transitions (ST) [16]. Within a few decades, international communities and institutions have been developed to foster this research field, along with several representative theories and methodologies being proposed. Sustainability Transitions Research Network (STRN) is inaugurated in 2009 and has been one of the most influential international research communities that broadens the understanding of ST research from both macro-level and micro-level, from grand nature-society interactions to individual attitudes and motivations. To be more specific, nine themes of transitions research are summarized by STRN, namely, (1) Understanding transitions; (2) Power, agency, and politics in transitions; (3) Governing transition; (4) Civil society, culture, and social movements in transitions; (5) Organizations and industries in ST; (6) Transitions in practice and everyday life; (7) Geography of transitions: spaces, scales, places; (8) Ethical aspects of transitions: distribution, justice, poverty; and (9) Methodologies for transitions research [17].

This renewed research agenda is first initiated in 2015 at the 6th International Sustainability Transitions Conference, and then gets refined and finalized as a research report in 2017. Based on this report, a modified article which further reviews the current state of transition research and meanwhile calls for continued efforts is published in a prestigious journal in 2019 by Köhler et al. (2019) [18]. This innovative regime of Köhler et al. provides a more comprehensive and systematic method, to scan and tackle both the academic controversies and practical themes in detail. After its publication, it immediately gets enormous attention both within and beyond academia; soon it has been borrowed into analyzing various research topics and disciplines, such as sustainable business model innovation [19], transformational social change [20], and social movements and institutional entrepreneurship [21].

In addition, other frameworks in transition studies are emerging from time to time which also provide nuanced perspectives into viewing sustainability research and practice. These emerging frameworks can be found as transition management in the policy implementation sphere [22, 23] strategic niche management aims to design instruments and experiments to govern transitions into socially and technically desirable directions [24, 25]; multi-level perspective analyzes broader problems on socio-technical transitions [26, 27]; and technological innovation systems provide practical guidelines for policy-making [28]. These abovementioned frameworks mainly lay focus on one or few specific perspectives, thus still lacking a comprehensive outlook to cover complicated large-scale transformations as detailed as the nine themes provide. Therefore in this paper, by referring to sustainability transitions, we are mainly positing e-commerce research under the aforementioned nine themes, to investigate how tightly e-commerce has approached ST prospects proposed by leading researchers of STRN. By applying the multifaced nine themes, we can effectively cover the multidimensional e-commerce research as exhaustively as possible. The nine themes include the general understanding of sustainability, and more nuanced perspectives including politics and governance transitions, cultural, industrial, geographic, ethical, methodological aspects, and transitions in practice and everyday life. This ST regime is the theoretical framework in our research to classify scholarly papers at hand. In the next chapter, we will discuss e-commerce contexts under these nine themes in detail.

### Sustainability Transition and E-commerce

A prevailing understanding of ST normally refers to the fundamental and multi-dimensional transformation of large socio-technical systems moving towards more sustainable production and consumption modes in the long run. Production and consumption are among other grand challenges related to unsustainable patterns in social-technical systems, which cannot be achieved through incremental improvements but need shifts to new systems [29, 30]. E-commerce is tightly participating in the production and consumption process as prior studies already unveiled, such as in the food consumption and production industry [30], purchasing and production cycles [31], packaging production and use [32], and supply chain [33]. To take the food industry as a concrete example, information and communication technologies (ICTs) and electronic business-to-business data exchange interfaces (EDIs) have eased the process of the production and processing of food, to better meet consumers’ expectations for high product quality, and their expectation of food being produced in a sustainable way [34].

As in the consumption end, technological advancement in e-commerce reshapes how companies interact with their consumers and dramatically impacts the service creation process [35]. The easy communication and information flow increase sales of various products and provide a more convenient way of receiving services and products [36]. Also, e-commerce facilitates communication between customers and companies and provides a more feasible approach to encourage consumers to co-create value [37]. Thus, companies are better able to meet consumers’ demands while keeping surplus inventory to a minimum [38]. The existence of e-commerce itself represents a radical transformation throughout the production and consumption journey, as e-commerce embeds more information flow, and rewrites the traditional way of how production can be processed and consumed. More importantly, e-commerce has a great potential to reduce energy consumption [39, 40] and therefore has the capability to lead a more sustainable promise.

Under this premise, keen interest has been extensively growing in both sustainable future and transition management in the e-commerce domain. Recent studies have partially explored sustainable e-commerce which concentrated on packing materials, logistic management to reduce carbon emissions, and reducing negative environmental impact. Previous discussions of sustainable e-commerce have been built around environmentally friendly context and resource utilization perspective, a practical framework that enables the assessments of e-commerce performance under key elements of ST is still lacking, and the interaction between e-commerce and ST philosophy has been under conceptualized as well. Positing e-commerce research under the ST scope offers us a radical typology to understand fundamental sustainable elements in the e-commerce domain and precisely aims to understand the dialectic relationship between stability, path dependence, and change in multiple facets. To conduct a comprehensive literature review incorporating ST theory to unveil yet-to-be-discovered themes and solutions hidden behind the e-commerce dilemma is therefore indispensable.

## Methodology

### Data Collection

This research aims to investigate current trends, research topics, and the stage of e-commerce research thoroughly. An academic database that covers reliable publications as much as possible will help us to systematically sample the literature and target high-quality journals. We adopt Scopus (www.scopus.com) to collect academic literature as Scopus has greater coverage of international journals in social science [41]. The original strategy attempts to contain as many articles as possible in the initial stage and then exclude unrelated papers by limiting the scope to the research questions at hand. The research terms are carefully chosen to cover both the sustainable and e-commerce domains. Search strings used in the Scopus database are TITLE-ABS-KEY (sustainable e-commerce or sustainable electronic commerce or e-commerce sustainability) AND (LIMIT-TO (DOCTYPE,”ar”)), which are in line with the main theme of the research. Content analysis is adopted to analyze the content of the sample articles. The actual analysis is done using Mendeley, which is a reliable and widely applied software to manage references. Full-text of the article is downloaded and imported into Mendeley; notes are made in it directly while reviewing the papers.

The first search shows 261 hints altogether (accessed in April 2022). We exclude conference papers first considering they have inconsistent quality; therefore, we limit our inquiry only to journal articles. Ninety-five conference papers are excluded and 136 journal articles are left. The second-round selection aims to exclude articles not written in English. We also exclude articles that are missing full text or cannot be downloaded. The last selection proceeds to see if the remaining articles are highly related to sustainable e-commerce contexts. In this round, 78 papers are excluded, and only 58 papers are left for further classification.

The main reasons for a decline in the papers’ quantity are that conference papers consist of a large proportion with comparatively inconsistent quality, and a large corpus of papers does not focus on the e-commerce domain but rather on smart cities, mobile phone life cycles, electronic equipment waste, etc. The initial result shows that an increasing amount of attention has been given to the sustainable e-commerce areas of international conferences and publications. Besides, only a small portion of papers is directed to multi-dimensional facets of sustainable e-commerce topics.

### Content Analysis

While the classification might be subjective and dominated by the main author’s preconception, a consistent methodology is needed to mitigate possible bias. Content analysis is a research method that provides an objective and systematic technique to describe and quantify valid inferences from visual, verbal, or written texts, into data that can be summarized and compared [42]. Applying content analysis allows us to maintain the research focus during the analysis process and ensure the validity and reliability of the following steps. Based on the hands-on guide to conducting a content analysis by Erlingsson and Brysiewicz [43], we strictly read the contexts and then start dividing up the content into meaning units. While reading the whole paper, we pay special attention to the theoretical background and methodology section; each paper is preliminarily classified according to the foundational approach and theory used in the paper. After this, the 58 papers are further labeled with codes and grouped into nine separate categories based on how the approach fits the core constructs of the nine ST themes mentioned above. We scan papers twice to ensure the consistency of the classification process. The stability indicates a high degree of reliability and repeatable results [44], which therefore confirms the validity of this study.

### Descriptive Results

In this section, we first offer a general analysis of the 58 papers collected at hand to give a glimpse of current topics and trends, and gain an overview of the current knowledge accumulation in the field of SE. Then, to further study their inner logic, papers are categorized into the abovementioned nine themes based on the content analysis method.

**Fig. 1:**
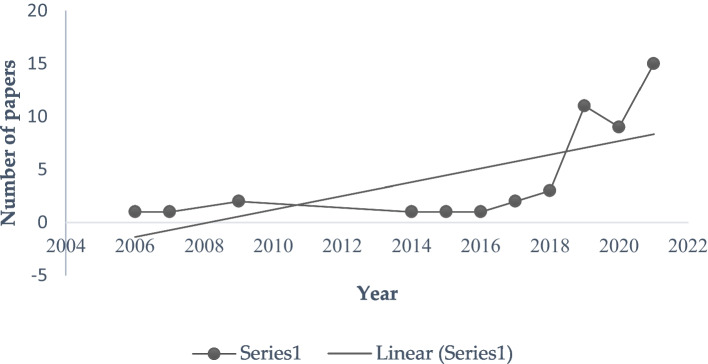
The number of research articles published in the Scopus database. Source: own elaboration

Figure 1 displays the number of papers published in the Scopus database across the selected study period. The 58 articles span from 2006 to 2022. We do not limit our research to any specific time so we can include all papers since the very first publication regarding sustainable e-commerce research, which is published in 2006. A general increasing trend in the number of academic papers confirms an escalating interest in sustainable e-commerce research. The sharp acceleration appears after 2019; 46 papers are published in the last 3 years, occupying 79.3 percent of all papers that have been published from 2006 to 2022. This result shows a sharp escalation of interest in the SE domain which indicates that this research domain is relatively new but gaining ample attention from academia throughout the last 5 years.

We also take a brief overview of the citation times of each paper. Among the 58 papers, the most influential paper has been cited 41 times, and 15 papers are yet to be cited. Twenty-seven papers have been cited between 1 and 10 instances, which occupies 46% of the collected database of papers. This result shows that a large proportion of SE papers have relatively limited influence and have not yet been disseminated widely.

Through keywords, we also get to know which industries gain researchers’ attention. Apart from three papers lacking keywords, “delivery” and “logistics” each appears 16 times respectively, indicating that in the SE- related topics, delivery, especially “last-mile delivery,” has been a hot topic. Researchers are trying to propose solutions and designate algorithms to alleviate pollution caused by e-commerce shopping activities. “Policy” follows 6 times and “business” appears 7 times, as does “consumer,” meaning that sustainable e-commerce has multidimensional contents intertwined, and the topic of sustainable e-commerce covers several disciplines, such as policy strategy, consumer relationship, business, and management.

## Results

### Literature-Driven Classification Based on ST

As we mentioned before, to gain a deep understanding of how the existing research topics are scattered alongside various directions, here we borrow nine themes identified by Köhler et al. (2017) that address different aspects of ST research. We classify articles collected beforehand and investigate to what extent each paper fits into these nine themes by identifying their research contents, research design, etc. We code the main themes, main concepts, and keywords, then fit them into Köhler’s research regime. Eight themes have been identified, there is no paper detected under the theme of “Governing transitions.” The coding results of the collected 58 papers are presented in Table 1; each theme is elaborated on in the following context.

**Table 1:** Paper divisions are guided by the ST regime

Themes	Main concepts	Keywords	Reference
Understanding transitions	1) Possibilities to improve sustainable last-mile transport in an omnichannel environment 2) A literature review to understand sustainable last-mile delivery in the e-commerce market 3) How the antecedents of e-loyalty affected consumer perceptions of e-commerce sites 4) An understanding of the net environmental sustainability of shopping 5) How incentives (non-financial) drive e-commerce delivery in a sustainable way 6) Understand the contribution of eBay users of trading used goods online to resource efficiency 7) Factors influencing consumers’ impulse buying behavior 8) Understand if product information increased sustainable purchases 9) Understand private labels on selecting sales mode 10) An understanding of households’ shopping and travel styles in the USA 11) How service value has been co-created on an e-commerce second-hand platform	Last-mile delivery, stakeholders, consumer behavior in e-commerce, E-loyalty, website accessibility, omnichannel retail, online marketplaces and online shopping, sustainable consumption	[45–55]
Power, agency, and politics in transitions	1) A policy framework for sustainable cross-border e-commerce in China 2) Minimize expected economic costs and cut down the environmental impact by various policies and sustainable strategies 3) Inventory share policy for a sustainable omnichannel e-commerce network	E-commerce, resilient policy, supply chain, sustainability, uncertainty analysis	[56–58]
Governing transition	No paper was detected		
Civil society, culture, and social movements in transitions	1) From the Chinese cultural concept and social trust (*guanxi*) to analyze if the web marketing mix is sustainable 2) Use digital social innovation (DSI) to address societal challenges and analyze how grassroots communities leveraged e-commerce to overcome poverty	E-commerce, social trust, digital resilience, China, sustainable development goals, digital social innovation, technology leapfrogging	[59, 60]
Organizations and industries in ST	1) Comparative analysis of traditional and e-commerce DVD rental industry from energy, environmental, and economic aspects 2) An approach to sustainable e-retail in the lingerie industry 3) Sustainable logistics correlated to customer satisfaction and repeat purchasing behavior 4) Sustainable logistics service in the fresh food e-commerce industry 5) A comparison of electric vehicles in last-mile deliveries in business-to-consumer (B2C) e-commerce 6) Adopt the deliver‐from‐store model and the impact on supply chain members’ sales model options 7) The home-delivery agri-food supply chain in the emerging e-commerce market 8) A research framework focuses on the logistics perspective 9) Sustainable e-commerce in the apparel industry	Industrial ecology, sustainable consumption, E-commerce, Internet marketing, SMEs, customer satisfaction, purchase intention, fresh food e-commerce, last-mile logistics, environmental sustainability, channel strategy, omnichannel, cooperation stability control, strategic alliance, supply chain extension, service-dominant logic	[61–69]
Transitions in practice and everyday life	1) A multi-objective optimization model to achieve sustainable reverse logistics in the e-commerce market in India 2) Second-hand products selling practice	Multi-objective, reverse logistics, sustainability, E-commerce, consumer behavior, ethical practice	[70, 71]
Geography of transitions: spaces, scales, places	1) Potential reductions in carbon dioxide emissions from passenger transport in Sweden 2) An analysis of pick-up point networks in Antwerp, Belgium 3) Outdoor decking material selection in the e-commerce market in Finland 4) Norwegian females’ environmental attitudes and behaviors for last-mile delivery options 5) Compare different deliveries for online ordered non-food products in Belgium, in three area types 6) Sustainable e-commerce competition in Romania	Online shopping, transport policy, home delivery, urban logistics, consumer choice behavior, environmental communication, sharing economy, sustainable last-mile delivery, market share, sustainable competitiveness	[72–77]
Ethical aspects of transitions: distribution, justice, poverty	1) Influence of sustainable marketing on customers' word-of-mouth concerning online retail 2) Liability and security breaches in mobile payment for a sustainable e-business 3) Adoption of proactive fraud detection strategies for data security 4) A trust-embedded contract for sustainable information sharing 5) Research on the security of payment, personal data, payment cards, and purchased goods	Affective commitment, customer satisfaction, E-commerce, electronic word-of-mouth metrics, sustainable behavior, fraud transactions, liability, payment systems, anomaly detection, business intelligence, information sharing, regulation, e-customer, security, social threat, sustainable development	[78–82]
Methodologies for transitions research	1) An agent-based model to understand the environmental impacts of traditional bookstores, self-pickup options and e-commerce 2) Elements for a sustainable e-business model 3) A conceptual content model to enhance the website for a renewable energy source enterprise 4) Using fuzzy cognitive maps and value-focused thinking to achieve sustainability in e-commerce channels for additive manufacturing 5) A sustainable networked delivery system 6) Crowdsourced delivery as a tool to mitigate problems caused by the last-mile city logistics 7) A path analysis model to investigate the quality of e-commerce 8) Using blockchain technology to decentralize the accessibility of e-commerce products 9) Fuzzy AHP and interpretative structural model to learn the influencing factors of rural last-mile delivery 10) A hybrid fuzzy multicriteria decision-making approach to assist e-commerce businesses 11) New business models applied through contextual ambidexterity 12) Session-Based Recommender system for sustainable digital marketing 13) A mixed-integer nonlinear programming model in a B2B e-commerce platform for multi-product delivery operations 14) A new mixed distribution model for e-commerce last-mile deliveries 15) A concurrent mixed method to explore sustainable value creation in the Enugu state 16) A methodology for choosing the most sustainable deliveries on last-mile logistics 17) A methodology to decide on city logistics center and vehicle routing problem 18) A framework to guide managers in sustainable decision makings in a supply chain context 19) Using the PLS-SEM Methodology to examine the determinants of cross-border cooperation 20) Setting a business model to improve e-commerce sustainability	Multicriteria method, sustainability, E-commerce, business model, strategic planning, value-focused thinking, modeling, sustainability influencing factors, decision making, business model innovation, design science research, value creation, analytical model	[83–102]

Source: own elaboration

Under the theme of “Understanding transitions,” eleven papers are detected. Articles within this scope are mainly probing into a general understanding of sustainable e-commerce, either delineating possible factors affecting e-commerce sustainability or what effects e-commerce might bring to the environment, for instance, the environmental impact of purchasing item(s) online [45], and the possibilities to improve last-mile transport in a sustainable way in an omnichannel environment [46]. Possible environmental premises that lead to sustainable practice in certain industries are being researched, for example, sustainable e-commerce delivery [47] and last-mile delivery in cities [48]. Researchers are striving to understand incentives affecting consumers’ attitudes by conducting literature reviews or empirical analyses. Two papers identified are centered on consumers' perceptions. One paper focuses on how sustainability of e-business affects consumers’ attitudes [49], while another paper explores the contribution of eBay users trading used goods online [50]. Last-mile delivery, omnichannel retail, and transportation are frequently used as keywords within this theme.

Suggesting or implementing sustainable frameworks, policies, strategies, and structures are the trending research topics found in the “Power, agency and politics in transitions” theme. Researchers and practitioners propose solutions by combining theoretical background with specific questions, to tackle practical sustainable issues within certain contexts and industries, such as “cross border e-commerce [56],” “supply chain,” and “waste management.” Although those research topics are concentrating on strategic policies, the realization of these abovementioned policy proposals are relying on multiple factors such as “omni-channel [57],” “hybrid strategy,” and “supply chain extension [58].” This result indicates that achieving a sustainable future in multi-dimensional domains cannot solely rely on policies as they are not sufficient, but rather both top-down and bottom-up participation are required. Three papers collected can be broadly classified as general policy designs which are mainly advocating and suggesting political frameworks, rather than implementing practical policies by the administrative government departments.

No paper is found within the scope of “Governing transitions,” which indicates a comparative deficiency in this specific topic area. Governance in the sustainable development context is about encouraging shifts toward more environmentally sustainable development by reforming practices of socio-political governance [103]. Governance often plays a particular role in ST, which has been exercised through relatively stable sets of norms, rules, and practices that prioritize public issues and impose and implement decisions on them [104]. A long-term policy design, so-called reflexive governance, brings radical change to key societal structures and innovates new socio-technical systems of provision [105]. Therefore, governing transitions and relevant governmental guidance in the ST topics should have received more concern, but unfortunately, we could not detect any papers within this particular scope.

Only two papers are categorized into the “Transitions in practice and everyday life” theme, in which Dutta et al. (2020) suggest adopting better practices for sustainable reverse logistics in the e-commerce market. Arman and Mark-herbert (2022) conduct interviews to analyze sustainable reselling practices in users’ everyday lives. This very limited finding may indicate that even though e-commerce has widely penetrated people’s daily life, however, the awareness of more sustainable e-commerce is yet to have materialized. When comparing other former research findings regarding transition practices, we notice that Geels et al. (2015) define “reconfiguration,” in their work which focuses on socio-technical systems transitions and practices in daily life. Also, Hyysalo et al. (2013) detect Finnish energy end-users and their inventions to modify renewable energy technologies in practice. By comparison, research topics such as in the sustainable e-commerce practice domain are still lacking, leading to our call for a more nuanced understanding based on further exploration.

Two papers are found under the umbrella of the “Civil society, culture and social movements in transitions” theme, with one attention paid to the Chinese cultural concept of “guanxi [59],” while another paper addresses societal challenges and analyses how the remote grassroots communities leveraged e-commerce to get out of poverty [60]. Both papers discuss the advantages of digital marketing and e-commerce from a certain cultural and social context, and particular attention is given to specific cultural contexts. Useful insights gained from these two papers for other countries to borrow are also summarized.

A distinct characteristic of the theme of “Organizations and industries in ST” is that topics are centered on certain domains and industries and collaborative efforts are suggested to achieve a sustainable premise. The fashion industry, logistics industry, and supply chain are the main areas that researchers focus on, and small to medium-sized enterprises are the major participant party suggested to take part in the ST tide. Various structures, strategies, and approaches have been carved out to advocate active collaborations between multiple departments, which can be found in Han et al. (2020), Zheng and Wang (2021), and so on.

Regarding the “Geography of transitions: spaces, scales, places,” six papers are included in this theme, with all papers investigating sustainable-related topics in European countries, and three papers are conducted within Nordic countries, including Finland, Sweden, and Norway. This result suggests that the sustainability issue is gaining more attention in such developed economies as Sweden, Belgium, and Norway, but lacking discussion within emerging economies and Asian countries, such as China and Korea. These six papers limit their research background and settings to exclusive geographic scales and only demonstrate sustainable e-commerce topics within certain spaces; therefore, they are categorized in this group.

In the theme of “Ethical aspects of transitions: distribution, justice, poverty,” we find five papers that lay focus on data security in e-commerce payment, fraud detection systems, trust contracts, liability and personal data security, and other ethical topics. Under this theme, concrete strategies, contracts, and regulations are proposed by researchers to create a more reliable online shopping environment. Five papers can be further divided into two types, elaboration of ethical issues and proposals of proactive strategies. On the one hand, Anastasiei and Dospinescu (2019) predict affective commitment influences electronic word-of-mouth, and reach a conclusion that commitment and satisfaction have a significant impact on both word-of-mouth volume and valence for online retailers. Pabian et al. (2020) highlight the security of the means of payment, personal data, payment cards, and purchased goods. On the other hand, Saia and Carta (2019) suggest proactive fraud detection strategies benefit big data information security. Han and Dong (2017) also introduce a trust-embedded contract in e-commerce content; its impact and effectiveness of the regulation mechanism have been highlighted in their paper. Consumers’ satisfaction and commitment, regulations, and trust are frequently mentioned as keywords within this theme. Maintaining a dynamic relationship with customers and improving customer satisfaction and mutually achieving a secured sustainable e-commerce development is detected as the main goal under this theme.

Twenty academic papers belong to the category of “Methodologies for transitions research,” which occupies the largest proportion of the whole database. Research topics are varying under this theme but all papers are forming conceptual frameworks or analytical/empirical models to mitigate the problems emerging from the unsustainable supply chain, production, energy consumption, resource inefficiency, and logistics of delivery. Massive research finding on this topic indicates that researchers are keen to figure out the main obstacles and problems that hamper sustainable development, and practical solutions are tested through modeling and theoretical hypotheses. Both quantitative and qualitative analysis can be found within this category and a large corpus of practical suggestions can be built upon to solve existing sustainability issues. Feasible scientific outcomes provide concrete solutions for e-business enterprises. Some of them include strategizing sustainability in e-commerce channels by using fuzzy cognitive maps and value-focused thinking [83], a conceptual content model to enhance the website for a renewable energy source [84], a crowdsourced delivery tool to lessen the problems resulting from last-mile city logistics [85], etc.

### An Integrative Perspective of Sustainable E-commerce Research Based on ST Logic

In this section, we present how the already defined nine themes and papers fit the conceptual definition of ST ideology. The logic here is to first compare our previously detected papers with the core ideas extracted from the ST agenda, and analyze the missing parts that current academic research lacks (Table 2). Our unique contribution here is that we propose possible theories and ideologies to fill in the gap that the current research has not reached, and suggest future directions that need further development. By reviewing the existing literature, we have a broad picture of how e-commerce and ST have been intertwined, and therefore be able to introduce relevant and applicable theories to fulfill the urgent need for a breakthrough in e-commerce research.

**Table 2:** Research gaps on ST logic and possible research directions

Themes	Core idea of ST ideology is borrowed from Köhler et al. (2017)	Main concepts detected from papers collected	Concepts missing and possible future directions
Understanding transitions	Various perspectives and approaches to capture the key phenomena and pathways of ST	Understand the possibilities, effects and antecedents of sustainability in the e-commerce environment	A systematic framework to thoroughly understand the whole picture of ST in e-commerce development
Power, agency, and politics in transitions	Content of public policies, process, outcomes, how policymakers are involved in policymaking, notion, and power	Implementation of policy framework, strategies, and policy designs	Policymakers’ and stakeholders’ perspectives, their capabilities, and how to apply efficient policy to affect public awareness, and eventually lead to solid transitions in the e-commerce context
Governing transition	Governing transitions not only from a top-down perspective but also with the private sector and multiple actors to solve societal problems	No contents detected	To remove the ambiguity of governance among public administrations, and encourage cross-sector cooperation. “Transition management” [106] can be borrowed. Another related theme is Social Innovation (SI)
Civil society, culture, and social movements in transitions	A wider range that not only includes public and private sectors but also organizations to bring social, cultural, and industrial transitions	Mainly cultural content and societal challenges are mentioned	A broader scope of public awareness is missing, also lacking an understanding from the organizations’ point of view
Organizations and industries in ST	How industries and institutions united to make creations and shape transformation broadly in societal, political, and institutional discourse	Encouraging new business models and strategies, suggest tighter cooperation within certain industries	The inability to legitimate industry standards, and solutions raised by researchers are not reaching a political level and so lacking convincing restrains. “Sustainable industrialization” [107] and cross-sector collaboration [108] might be a direction
﻿Transitions in practice and everyday life	Approaches that view the whole production-consumption chain, and investigate causal interactions that result in everyday consumption	One paper focuses on the daily practices of a singular section	Interactions between consumers are missing. “Value co-creation” [109] and interactive engagement with customers might be a starting point. Social movements such as “FridaysForFuture” that have received public recognition and influenced public discourse [110] need more attention
Geography of transitions: spaces, scales, places	How transitions differ or are similar in various locations, and explaining how place-based factors enable and constrain the differences or similarities	The current state of ST in a defined geographic area	Factors influencing similarities or differences in a certain geographic area, also lack in how factors are influenced by geography
Ethical aspects of transitions: distribution, justice, poverty	Notions of equity and justice, human rights, well-being and social welfare, relations between humans and the natural world and how to distribute natural resources	Ethical interactions between consumers and online systems	Future research can consider welfare, resource distribution, equality, and justice. An ethical framework contains both classic and practical principles that can be borrowed; see example [111]
Methodologies for transitions research	Methodological approaches to investigate, solve, or propose solutions	Solutions, methodologies, models	Normally focus on in-depth single cases and therefore lack institutional theory. Socio-technical transition (STT) perspective can integrate technologies, actors, networks, and multiple sectors to assume interplay roles

Source: own elaboration

The main findings worth mentioning here are lacking in the governing transition section with no contents detected, leaving an appeal for more future attention. Also of interest is that both in the general understanding of ST and as pertains to social and cultural movements, a broader and joint consensus about e-commerce’s sustainability is needed. To achieve tighter cooperation on the sustainable e-commerce issues between various industries, agreeing upon standards that are beyond disputes and private interests is needed, and we suggested borrowing “sustainable industrialization” and also advocated cross-sector collaborations to achieve a mutual goal. In the theme regarding “transition in practice and everyday life,” we detected that engagement and interactions between firms and consumers are lacking.

There is a need to encourage the public to both gain awareness of sustainability concepts and also to get engaged in sustainable practice. We suggested the concept of “value co-creation” that encourages the customer to interact dialogically with service providers [109]. E-commerce provides an advantageous opportunity in information exchange to facilitate this process.

## Discussion and Conclusions

Motivated by the booming escalation of e-commerce research in recent decades, especially since the beginning of the covid-19 pandemic, an intense discussion has been molding the e-commerce research both in academia and in practice. To keep e-commerce developing towards an economic-societal friendly path and to fit a sustainable future, it is observable that many e-commerce marketplaces are cumulatively introducing sustainability strategies [99]. This paper aims to analyze existing research trends within the scope of sustainable e-commerce by introducing the ST continuum into coding 58 academic papers at hand. This research explores the current state of the trending research themes, identifies missing topics in the scientific papers, and suggests future directions for researchers and policy-makers to borrow from. Three research questions have been answered and the major findings of this study can be summarized as follows:

First, this study manifests that there is a sharp escalation of interest in the SE domain; however, this leads to overwhelmingly intertwined and overlapping concepts in the sustainability research that are rather confusing; there is also a lack of systematic approach to synergize multi-disciplinary studies. It is difficult to find one unified theory to integrate dispersive multi-disciplines, and therefore the proposal from academia does not quite penetrate various industries, not to mention achieve fit in diverse scenarios. In that light, the innovations this paper reiterates are twofold. For one thing, our approach provides a replicable method to posit academic studies under an insightful regime to extract the main ideologies existing in the current state of SE research, to achieve synchronous understanding and action on SE matters from multi-dimensional sectors. For another, this research proposes complementary theories to compensate for the relatively lacking theory. Necessary approaches such as “omnichannel” or “hybrid push–pull strategy” have been advocated to reach a cross-sector collaboration from an academic perspective. Extant theories and practices can be borrowed into fulfilling a hybrid strategy, to cover sectors from the input of resources to the end users. For example, “sustainable supply chain management” can be deployed to improve resource efficiency; it can also generate more awareness from consumers and encourage more sustainable e-commerce consumption.

Second, the existing literature shows an uneven focus on the nine dimensions of sustainability transitions research. Especially, this research identifies a lack of support to reflect on a governmental perspective on how to efficiently unite across sectors to achieve long-term sustainability goals. Sustainability transitions, by their nature, are coming along with tension and conflict, as the process is full of uncertainty and therefore needs specific implementation and incremental steps to reach radical, large-scale outcomes [106]. It is therefore a great challenge for policymakers to designate agenda that meets the requirements of such complex purposes. To achieve this goal, not only the lacking “governing transitions” research is urgently needed, but practitioners will also need to practice a more efficient governing strategy. “Transition management” can be regarded as a different type of governance model that develops interactive processes to gather networks of actors and to generate a broad “philosophy of governance” that makes the decision in uncertain conditions, also based on long-term goals. Introducing transition management ideology might compensate for the deficient practices directed to governing transitions and shed some light on a systematic innovation to achieve sustainability benefits.

From a practical point of view, there is a persistent lack of an industrial standard to guide various departments in one direction. This would be a most useful target of public policy. The current situation leads to loose restraints, scattered resources, and inferior multi-channel cooperation. There is an avid need to integrate all parties, including the public, policymakers, SMEs, stakeholders, manufacturers, laborers, and every participant to jointly devote themselves to supporting this surging radical change. Ultimately, both governance research and practice leave abundant room for improvement.

Third, this study also provides managerial implications for the companies. Companies can benefit from paying increased attention to ongoing themes in ST, because this arises their awareness of changing demands, and will ultimately affect their ability and competitive advantage to cater to demand. As a fledgling research area that deserves further attention, and indeed captures many important elements, ST can form another lever in the contemporary manager’s toolbox. This lever can exert the greatest benefit by also tying in research and practice from the rich and more established fields of social innovation and change management into pursuits of ST. Business and entrepreneurs must evolve together with society to ultimately fulfill their purpose of catering to society’s demand and its betterment. And there remains ample opportunity for first movers to shape that demand; to assume a proactive stance, rather than a reactive one that is left to play catch-up. Socially innovative offerings, under which the ST ideology falls, are ever more demanded by customers of the private sector and the public alike, yet supply-side uptake has remained limited [112], providing an opening for grabbing true competitive advantage.

While competitive advantage is often shaped by an idea or technology’s patentability and barriers to entry, early movers in new models and technologies collect benefits [113]. Most importantly, from a contemporary business perspective of high disruption and great uncertainty, being able to shape and then satisfy demand, for instance, through platform expansion and education and sequent value co-creation, reinforces a virtuous cycle. Businesses should elucidate a top-level strategy that is acutely aware of the ST frontier’s developments, and to this end might borrow from Mintzberg’s (1973) famous managerial modes of *entrepreneurship*, *adaptation*, and *planning* and find the right balance amongst each for all of the different phases of inevitably approaching ST paradigms—both endogenously and exogenously.

As this paper’s character leans towards a literature review paper, its depth and scope may be limited by the selected database and methodology applied. Even though we consider the Scopus database to have wide enough coverage of open-access papers, the singular selection may narrow down our conclusion. We would therefore suggest including other academic papers from other databases, for example, Web of Science and Google Scholar. Also, in line with the findings that ST is very much intertwined with policies, we would also suggest reviewing published official reports by intergovernmental bodies and organizations to widen the scope of this study.

Finally, even though we find the ST continuum is quite a comprehensive and applicable framework to help various industries scan their current status and the elements they may lack, we still haven’t found concrete practice employing this framework, especially from the e-commerce domain. How far ST might also lead to fundamental transformations in e-commerce has found little resonance. We therefore suggest that more attention be paid to this continuum, and more targeted practice be put into dealing with yet to be achieved unsustainability issues.

## Data Availability

Yes, the author can provide the data if needed.
